# A molecular dynamics study on the mechanical properties of Fe–Ni alloy nanowires and their temperature dependence

**DOI:** 10.1039/d0ra07831j

**Published:** 2020-11-03

**Authors:** Jianxin Chen, Pengtao Li, E Emily Lin

**Affiliations:** State Key Laboratory of Solidification Processing, MOE Key Laboratory of Materials Physics and Chemistry in Extraordinary Conditions, Northwestern Polytechnical University Xi'an 710072 China lipengtao_1985@163.com lipengtao@nwpu.edu.cn +86 29 88460499; School of Engineering and Materials Science, Queen Mary University of London Mile End Road London E14NS UK

## Abstract

Fe–Ni alloy nanowires are widely used in high-density magnetic memories and catalysts due to their unique magnetic and electrochemical properties. Understanding the deformation mechanism and mechanical property of Fe–Ni alloy nanowires is of great importance for the development of devices. However, the detailed deformation mechanism of the alloy nanowires at different temperatures is unclear. Herein, the deformation mechanism of Fe–Ni alloy nanowires and their mechanical properties were investigated *via* the molecular dynamics simulation method. It was found that the local atomic pressure fluctuation of the Fe–Ni alloy nanowire surface became more prominent with an increase in the Ni content. At low temperature conditions (<50 K), the plastic deformation mechanism of the Fe–Ni alloy nanowires switched from the twinning mechanism to the dislocation slip mechanism with the increase in the Ni content from 0.5 at% to 8.0 at%. In the temperature range of 50–800 K, the dislocation slip mechanism dominated the deformation. Simulation results indicated that there was a significant linear relationship between the Ni content, temperature, and ultimate stress in the temperature range of 50–800 K. Our research revealed the association between the deformation mechanism and temperature in Fe–Ni alloy nanowires, which may facilitate new alloy nanowire designs.

## Abstract 2

Deformation mechanism and mechanical property of Fe–Ni alloy nanowires are investigated through molecular dynamics simulation method.

## Graphical abstract

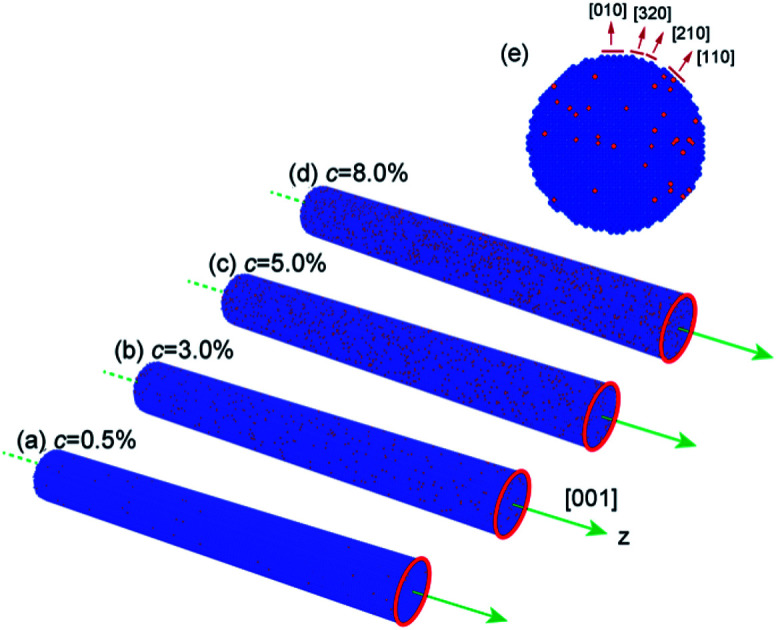

## Introduction

Magnetic nanowires (NWs) have attracted increasing attention for their distinctive properties^1^ (magnetic, electrical and mechanical) and show great potential in applications such as ultrahigh-density storage devices,^2^ probes,^3–5^ nanomotors,^6^ catalysts,^7–9^ and electrical devices.^10,11^ The design, manufacture, and application of NW-based devices are highly dependent on the mechanical properties of NWs, which are always significantly different from their bulk counterparts owing to their extremely small diameters and large surface-to-volume ratios. Besides, the quantitative characterization and analysis of the mechanical properties of NWs also shed light on the understanding of theoretical properties of the corresponding bulk material.

To date, several experimental methodologies, including atomic force microscope (AFM) and *in situ* electron microscopy, have been developed to characterize the mechanical properties of NWs.^12–18^ The AFM-based three-point bending technique has been developed and widely applied in calculating the elastic modulus of NWs.^19^ The radial elasticity of the NWs could also be calculated through the resonance frequency and deflection changes by a contact resonance AFM measurement.^20^ By integrating a nanomanipulator system such as AFM^21^ and a micro-electromechanical device^22^ with the electron microscope (SEM or TEM), the *in situ* mechanical testing of the NWs and real-time observation of the deformation process was achieved. Despite the rapid development of nano-dimensional manipulation and characterization techniques in the past few decades, the experimental characterization of the mechanical properties of NWs is still a big challenge due to its small dimensions. The test condition is limited and unable to evaluate the mechanical properties under extreme environment.

Computational methods such as molecular dynamics simulation, Monte Carlo simulation and discreteness simulation methods provide an atomic-level insight of the mechanical properties of the NWs.^18,23,24^ Molecular dynamic simulation is widely used to study the elastic modulus, deformation-mechanism and failure behavior of metal NWs, including Au,^25,26^ Ag,^27^ Ni,^28^ Fe,^29^ Cu,^30^ Mo,^31^ W,^32^ and Ti.^33^ As an important magnetic material, Fe-based NWs have attracted considerable attention. Choudhary *et al.* investigated the effect of orientation on the deformation of body centered cubic (BCC) iron NWs under tensile loading; the results indicated that the deformation mechanism varied with crystal orientation.^34^ The <100>, <112>, and <102> oriented NWs deformed predominantly by the twinning mechanism, while the <110> and <111> oriented NWs deformed by a dislocation slip. Temperature and size also affected the deformation mechanism. As in ultrathin <100> BCC Fe NWs, deformation by slip dislocation dominates at small sizes and high temperatures, while twinning is promoted at large sizes and low temperatures.^35^ Zhang *et al.* explored the influence of a 5-fold twin boundary on the structural and mechanical properties of face center cubic (FCC) Fe NWs.^36^ The higher Young's modulus and higher critical stress upon loss of elasticity were attributed to the twin boundary that suppressed the dislocation nucleation. Salje *et al.* reported the pseudo-elasticity and shape memory effect of α-Fe NWs under bending.^37^ They found that the nucleation of twins and nanoscale interfaces lead to pseudo-elasticity, which could be extended over a wide range of the wire diameter. Ackland *et al.* found that a totally different deformation mechanism applies for the tension and compression of the nanopillars of BCC Fe: dislocation glide in compression and twinning in tension, which is consistent with the experimentally-observed asymmetry in the nanopillar morphology.^38^ Dutta investigated the dislocation activities underlying the compressive deformation of BCC-iron nanopillars at a high strain rate. They found a direct correspondence between the bursts in dislocation activities and jerky deformation of the pillar.^39^ Temperature also has a pronounced effect on the deformation mechanism switch with the dislocation-plus-twinning mechanism shifting to completely dislocation-mediated mechanism as the temperature increases.

However, most of these simulation studies focused mainly on the Fe NWs and not enough attention has been paid towards the deformation behavior of the Fe-based alloy NWs.^40^ As an important noble-metal-free alloy, the Fe–Ni alloys have shown outstanding soft magnetic properties.^41^ So far, little is known about the role and mechanisms of Ni effects on the microstructure evolution in the Fe–Ni alloy deformation process. Herein, we report a systematic study for the effect of composition and temperature on the deformation behavior of the Fe_(1−*x*)_Ni_*x*_ alloy NWs using molecular dynamics simulation.

## The simulation methods

**Fig. 1:**
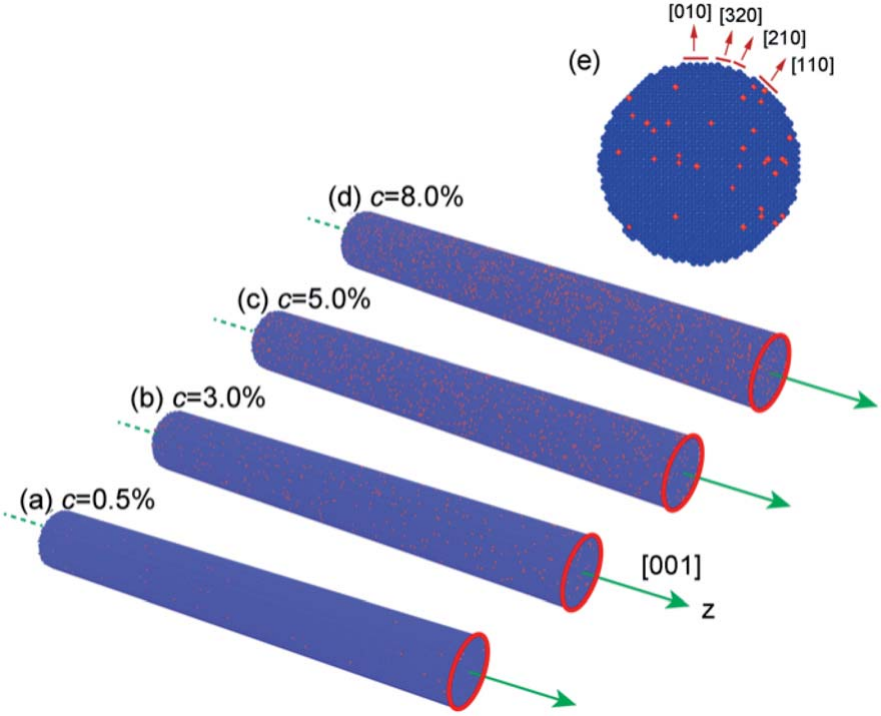
The simulation Fe–Ni alloy nanowire configuration (a) 0.5 at% Ni, (b) 3.0 at% Ni, (c) 5.0 at% Ni, (d) 8.0 at% Ni and (e) cross section of nanowire after energy minimization and relaxation. Fe: blue atoms, Ni: red atoms.

The molecular dynamics simulation was performed using the large-scale atomic/molecular massively parallel simulator (LAMMPS)^42^ with a constant time step of 3 fs. The NWs used in this simulation have a circular cross-section and a diameter of 8.0 nm, which were constructed from a single-crystalline BCC Fe–Ni alloy with different Ni concentrations (Fig. 1). A periodic boundary condition was imposed along the cylinder axis *z* ([001] direction) to mimic infinitely long wires, and the other two directions in NWs were kept free.^43^ The Fe nanowires prepared through the electrochemical deposition generally adopt a [110] orientation for its lower surface energy. However, [110] and [111] are the hard magnetization axis and [200] is the easiest magnetization direction. Numerous efforts have been made to control the direction of Fe nanowires and achieved great experimental success.^44^ Thus, in this work, we modelled the nanowires with the [001] orientation along the wire for its better magnetic property and application potential. The interaction between the Fe–Ni atoms was described by the embedded-atom-method (EAM) potential developed by Bonny *et al.*^45^ Maxwell–Boltzmann distribution was applied to initiate the velocities of the atoms. Also, the leapfrog scheme was used for integrating the Newton's equations of motion. Prior to tension loading, the NWs were subjected to energy minimization using the conjugate gradient method; heated up to the desired temperature in a step-wise fashion and relaxed in the Nose/Hoover isobaric–isothermal ensemble (NPT) for 1000 ps. During energy minimization and relaxation, the side surfaces of those nanowires evolve and form several kinds of faces (Fig. 1e). The tension strain was performed along the [001] direction at a rate of 8 × 10^8^ s^−1^ with canonical (NVT) ensemble.

## Results and discussions

### The local atomic pressure of the Fe–Ni alloy NW surface after energy minimization and thermal relaxation

**Fig. 2:**
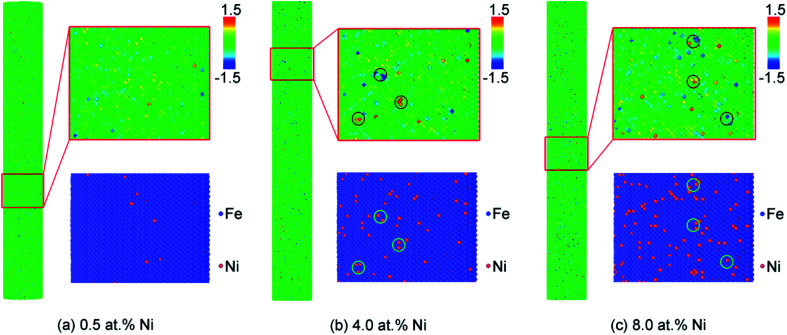
Local atomic pressure maps of Fe–Ni alloy nanowires with different Ni content (annealing at 300 K), (a) 0.5 at%, (b) 4.0 at% and (c) 8.0 at%. Atoms are colored according to the local atomic pressure. Pressures are given in GPa. The corresponding composition maps are shown in the below insert schematic. Fe: blue atoms, Ni: red atoms.

The local atomic pressure maps of the Fe–Ni alloy NW surface after energy minimization and system thermal relaxation (300 K) are shown in Fig. 2. The local atomic pressure is the stress an atom experience in the particular local environment and reflects how comfortable an atom is.^46^ In alloy, different topological and chemical properties of the alloy elements are the main origin of a local atomic pressure. First, the different atomic sizes of the alloy elements will affect their packing scheme and efficiency. It is well known that in a system consisting of two types of elements the large one prefers to locate on the nanoalloy surface, while the small one prefers to locate in the core, for the strain induced by the size difference between the atoms.^47^ Second, different chemical properties also influence the atomic-level stress state for their different bonding capability and charge transfer interaction. The Fe–Ni alloy showed an increasing local atomic pressure fluctuation with the increase in the Ni concentration for larger lattice mismatch. At a low Ni concentration (0.5 at%), there is no significant relationship between the local atomic pressure and the Ni atom distribution (Fig. 2a). This may be attributed to the similarity of the atom size and electronegativity between Fe and Ni. However, at a higher Ni concentration (4.0 and 8.0 at%), the local atomic pressure fluctuation becomes more prominent. Moreover, the sites where the atoms show a high local atomic pressure always have more than two Ni atoms in the surrounding (Fig. 2b and c). Those high energy sites may serve as the defect for dislocation nucleation.

### The influence of the Ni concentration on the plastic deformation mechanism of Fe–Ni alloy NWs

**Fig. 3:**
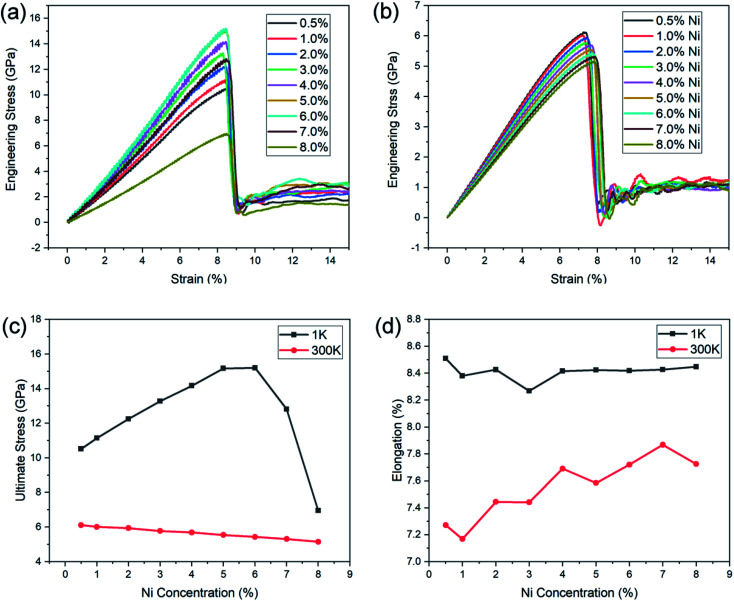
Stress–strain curves for Fe–Ni alloy nanowires at 1 K (a), and 300 K (b). Ultimate stress (c) and elongation (d) of Fe–Ni alloy nanowires with different Ni contents.

The stress–strain curves of the Ni–Fe alloy nanowires with different Ni concentrations (0.5–8.0 at%) under tensile loading are shown in Fig. 3.

The test was first carried out at low temperature (1 K) to eliminate the influence of thermal vibrations (Fig. 3a). The Young's modulus was obtained by calculating the slope in interval strain = [0, 0.03], in which the stress–strain relation was clearly linear for all the cases. Fig. 3a shows that all those alloy nanowires exhibit a similar stress–strain behaviour under uniaxial tension with a different elastic modulus. The stress increases with the increase in strain up to a peak stress, and then the yielding and breaking of nanowires lead to an abrupt drop of the flow stress. The Young's modulus and ultimate-stress increase at first with the increase in the Ni concentration (0.5–6.0 at%) and reach their maximum at a Ni concentration of 6.0 at%. Then, the Young's modulus and ultimate-stress decrease with further increase in Ni concentration (6.0–8.0 at%). The Fe–Ni alloy nanowires with a Ni concentration of 6.0% exhibit the highest Young's modulus of 181.5 GPa and ultimate-stress of 15.2 GPa. All those samples showed a similar elongation of 8.38–8.58%.

The room temperature (300 K) stress–strain behaviour of the Ni–Fe alloy NWs with Ni concentration in the range of 0–8.0 at% are shown in Fig. 3b. Similar to that of low temperatures, the stress of NWs at 300 K increases with the increase in strain and reaches its maximum, then drops drastically as the yielding and breaking of nanowires take place. The stress–strain curves clearly indicated that the increase in Ni has a solid-solution softening effect on the NWs; a similar result in the bulk iron alloy has been reported previously by Okazaki.^48^ The Young's modulus and ultimate-stress dropped when more substitutional Ni atoms were added (0.5–8.0 at%). Note that there is a linear relationship between the ultimate stress and Ni concentrations from about 0.5 to 8.0 at%. Also, the elongations increase slightly with the addition of Ni (1.0–8.0 at%). The significantly different influence of the Ni concentration on the Young's modulus and ultimate-stress at two temperatures suggests that there are different mechanisms dominating the tensile deformation.

**Fig. 4:**
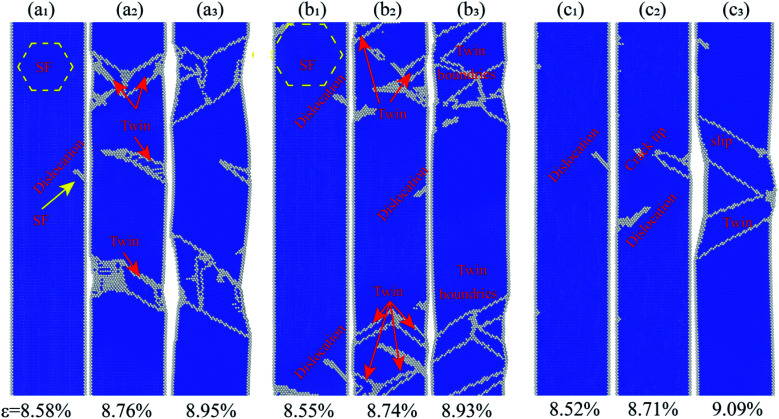
The atomic configuration of Fe–Ni alloy nanowires under different strain at 1 K. (a) 0.5 at% Ni, (b) 5.0 at% Ni, (c) 8.0 at% Ni.

In order to understand the influence of the Ni concentration on their deformation behavior, atomic configurations of the Ni–Fe alloy nanowires at different strain were analyzed. Fig. 4 shows the snapshots of the atomic configurations of the Ni–Fe alloy nanowires with different Ni concentrations ((a) 0.5 at%, (b) 5.0 at% and (c) 8.0 at%). It is well known that in body centered cubic (BCC) crystals there are two main plastic deformation mechanisms: deformation twinning and dislocation slip.^34^ It is reported that nanowire orientation, size, temperature and strain rate could affect the deformation mechanism of the metal nanowire.^34,35,49^ Herein, according to the features, the deformation mechanisms of Ni–Fe alloy nanowires vary with the Ni concentration. The Ni–Fe alloy nanowires with a low Ni concentration (0.5 and 5.0 at%) deform mainly by twinning mechanism under tensile loading. It has been observed that a twin embryo was generated on the free surface of the alloy nanowire (0.5 at% Ni) at the strain of 8.58% and nanowire began yielding (Fig. 4a). Then, the twin embryos develop into full twin, which was enclosed by two boundaries, with the increase in strain. Several other twins were also generated simultaneously from the free surface of alloy nanowire. The alloy nanowire with a higher Ni concentration (5.0 at%) also showed a similar behavior (Fig. 4b), but there were much more twins with smaller sizes. It is generally accepted that increased twin density results in the strengthening of materials for twin boundaries that suppresses the dislocation nucleation or hinder the movement of lattice dislocation.^36,50,51^ Combining atomic snapshots with the stress–strain curves, we can attribute the enhancement of the Ni–Fe alloy nanowires at low Ni concentration (>6.0 at%) to the twin induced enhancement. With the increase in the concentration of Ni, more defects were induced by the heteroatoms, which result in more partial dislocations to nucleate and emit form the free surface while yielding, followed by the formation of twin with small size. High density of the twin boundary results in strengthening of the nanowire.

**Fig. 5:**
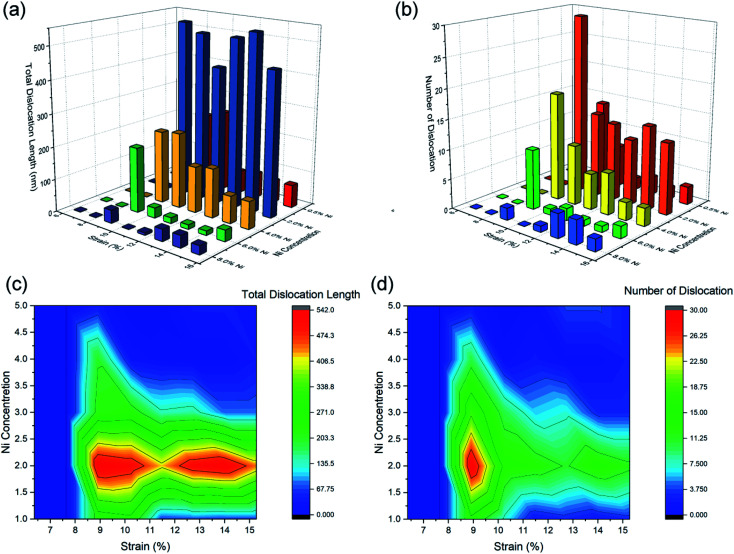
Dislocation length (a and c) and number (b and d) in Fe–Ni alloy nanowires under tension deformation at 1 K.

The atomic configuration of the Ni–Fe alloy nanowires with high Ni concentration (8.0 at%) was investigated to explore the decrease in the Young's modulus and ultimate-stress with the further increase in the Ni concentrations (6.0–8.0 at%). As shown in Fig. 4c, fewer dislocations and twins were found, the nanowire deformed through a dislocation slip mechanism. The dislocation extraction algorithm (DXA) analysis results of the Ni–Fe alloy nanowires under different strains at 1 K are shown in Fig. 5. It can be seen from Fig. 5 that the total dislocation line length first increases at the beginning of yield and then decreases during the loading process. The number and length of the generated dislocation in the alloy nanowires with a low Ni content (<6.0 at%) is larger than that in the alloy nanowires with a high Ni content (>6.0 at%). It is reported that in the Fe–Ni alloy, the stacking fault energy (SFE) decreases with the increase in the Ni element when the Ni content is below 36 at%.^52^ The reduction of SFE is in favour of the emission of partial dislocations from the nanowire surface and twinning formation.^53^ The increase of dislocation and twinning density leads to the strengthening of the material. However, further increase in the amount of the Ni element will result in dislocation formed in small stress and slip, leading to decreased strength.^54^

### The influence of temperature on the deformation of Fe–Ni alloy NWs

Temperature has a profound influence on the phases, microstructure and mechanical behaviour of metals.^55,56^ We also found different mechanical behaviours and deformation mechanisms in the Fe–Ni alloy NWs at 1 K and 300 K, as described in the previous section. Herein, we further examined the temperature dependence of the ultimate stress up to 800 K with nine different Ni contents to provide a deeper insight into the mechanical property of the Fe–Ni alloy NW.

**Fig. 6:**
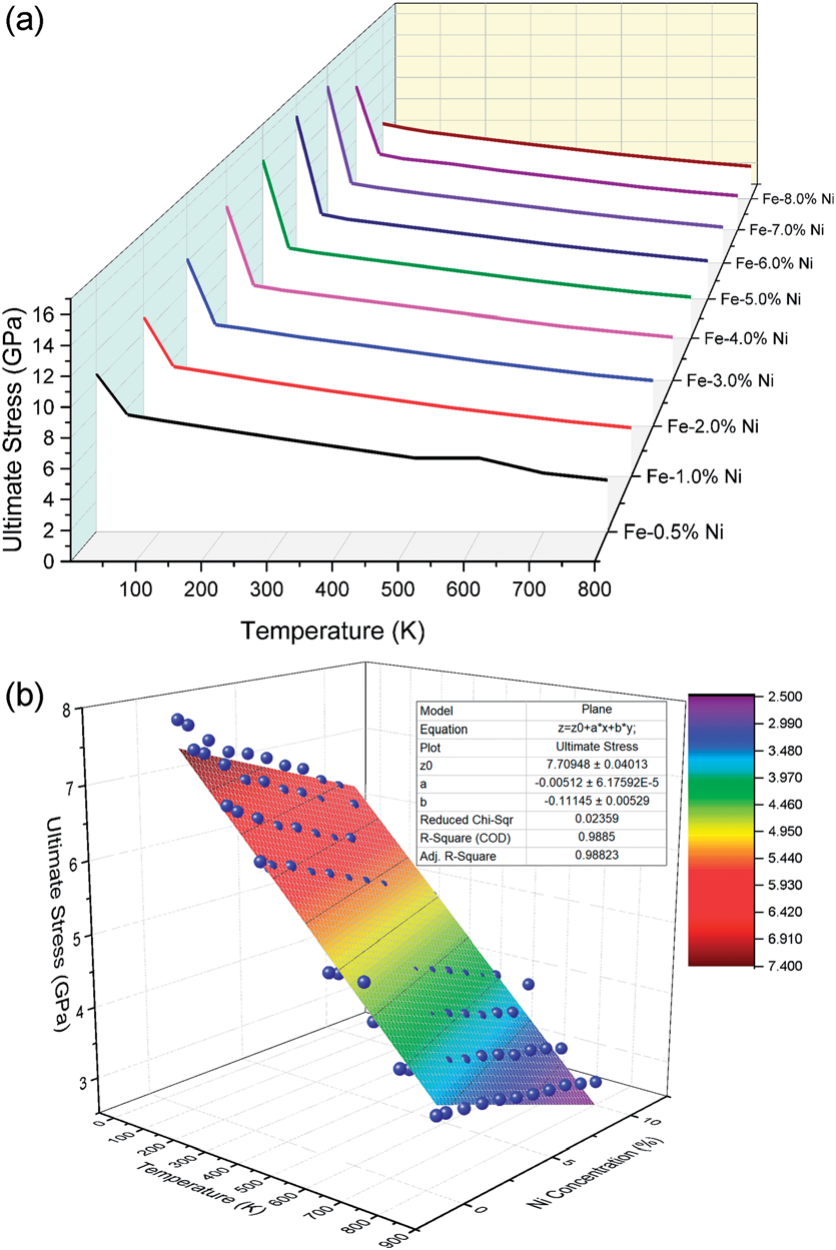
Images of the relationship of the ultimate stress, temperature and Ni content (a) and the fitted plane (b).

**Fig. 7:**
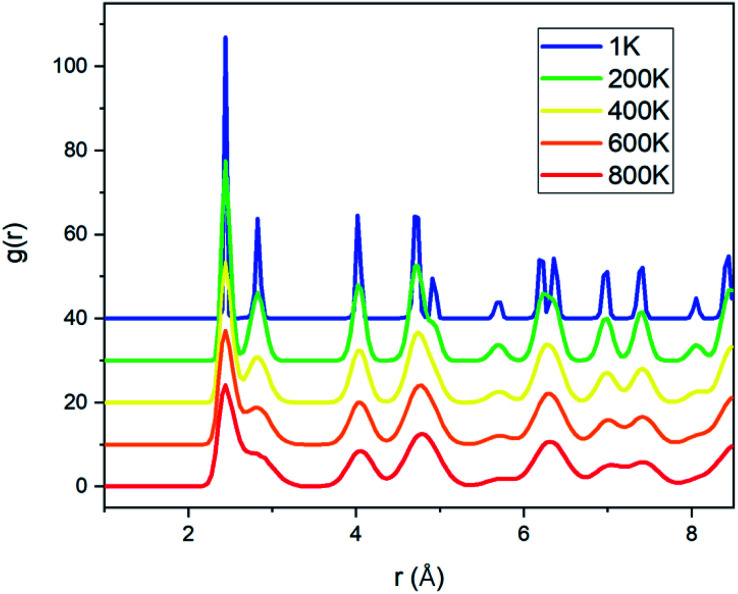
The radial distribution function (RDF) of Fe–Ni linking in NWs at different temperatures.

Fig. 6 shows that the ultimate stress of the Fe–Ni alloy NWs depends on the temperature. It can be observed that the ultimate stress decreases with increasing temperature. This may be because the stress for overcoming the barrier to plastic flow was reduced by the thermal fluctuation. The radial distribution functions (RDF) of the Fe–Ni linking in NWs at different temperatures are shown in Fig. 7. The width and height of the peaks of the RDF are related to the order of the micro-structure. As the temperature rises, the value of the RDF peak decreases and their width increases, indicating the increasing trend of thermal fluctuation and decreasing trend of the ordered degree. The higher the temperature, the lower the stress needed to overcome the barrier and start plastic flow.

Further investigation of the atomic configuration of the alloy NWs under tensile deformation at elevated temperature (50–800 K) showed that the dislocation slip is the dominant deformation mechanism in the Ni–Fe alloy tensile deformation at a high temperature (*T* > 50 K). The result is consistent with Choudhary's report that ultrathin <100> BCC Fe NWs deform by the dislocation slip mechanism at high temperatures while twinning dominates at low temperatures.^35^

The dependence of the deformation mechanism on the Ni content and temperature could be attributed to the stacking fault energy.

It is reported that for the alloys with small stacking energy twinning is more favourable to occur under external straining, and for the alloys with large stacking fault energy (>45 mJ m^−2^), the dislocation slip dominates the plasticity deformation of the alloys.^57–60^ With the increase in the Ni content or temperature, the deformation mechanism of the nanowires switch from twinning to dislocation slip.

Generally, the temperature dependence of yield stress (*δ*_Y_) has two trends: *δ*_Y_ = *A* – *BT* or *δ*_Y_ = *A* – *BT*^0.5^, where *A* and *B* are constants and *T* is the absolute temperature, depending on the interatomic potential model.^61^ Our simulation indicates that there is a linear relationship between the ultimate stress of Fe–Ni alloy NWs and temperature from about 50 K to 800 K (*δ* = *A* − *BT*), with a constant *B* of 0.00434, 0.00568, 0.00576, 0.00553, 0.00526, 0.00524, 0.00511, 0.00502, 0.00497, 0.00471 GPa and constant *A* of 6.30717, 7.94726, 7.84876, 7.64009, 7.39001, 7.30691, 7.14159, 7.0004, 6.87865, 6.64016 GPa for pure Fe, Fe – 0.5% Ni, Fe – 1.0% Ni, Fe – 2.0% Ni, Fe – 3.0% Ni, Fe – 4.0% Ni, Fe – 5.0% Ni, Fe – 6.0% Ni, Fe – 7.0% Ni, Fe – 8.0% Ni NWs, respectively.

Combining the linear relationship of ultimate stress-temperature and ultimate stress-Ni concentration, the influence of temperature and Ni concentration on the ultimate stress of Fe–Ni alloy NWs can been concluded as: *δ* = *aT* − *bC* + *Z*_0_, where *Z*_0_ = 7.70948 GPa, *a* = −0.00512, *b* = 0.11145 (*T*: absolute temperature, *C*: concentration of Ni atom).

## Conclusions

Through molecular dynamic simulation, the influence of the Ni concentration and temperature on the deformation mechanism and mechanical properties of BCC Fe–Ni alloy nanowires under tension loading were systematically investigated. The results indicated that the Fe–Ni alloy nanowires with a diameter of 8.0 nm deform by twinning mechanisms at low temperatures (*T* < 50 K) and low Ni content (*C* < 6.0 at%), while dislocation slip dominates at high temperatures (*T* > 50 K). The ultimate stress of the nanowires show a linear relationship with temperature and Ni concentration (50–800 K, 0.5–8.0 at% Ni).

## Conflicts of interest

There are no conflicts to declare.

## Supplementary Material
